# Seafood intake in children at age 7 years and neurodevelopmental outcomes in an observational cohort study (ALSPAC)

**DOI:** 10.1007/s00394-025-03636-7

**Published:** 2025-03-11

**Authors:** L. Nel, P. M. Emmett, J. Golding, C. M. Taylor

**Affiliations:** Centre for Academic Child Health, Bristol Medical School, University of Bristol, Bristol, UK

**Keywords:** Avon Longitudinal Study of Parents and Children, ALSPAC, Fish, Child development, IQ, Strengths and Difficulties Questionnaire, SDQ

## Abstract

**Purpose:**

Seafood is rich in other essential nutrients such as long-chain fatty acids, selenium and iodine that play an important role in neurodevelopment and cognitive function. The association between seafood intake in childhood and cognitive outcomes has not been well evidenced. Our aim was to investigate the association between seafood intake in children at age 7 years and cognitive and behavioural outcomes at age 7–9 years.

**Methods:**

Data on seafood intakes were collected at age 7 years in children enrolled in the Avon Longitudinal Study of Parents and Children (ALSPAC). Adjusted logistic regression was use to model seafood intake with the odds of suboptimal behavioural scores (Strength and Difficulties Questionnaire (SDQ)) measured at age 7 and 9 years and cognitive scores (IQ) at age 8 years.

**Results:**

Lower seafood intake at age 7 years (0 vs. ≥ 190 g/week) increased the adjusted odds of suboptimal prosocial behaviour measured by the SDQ at 7 years by 35% (OR 1.35 (95% CI 1.10, 1.81), *p* = 0.042) and at 9 years by 43% (OR 1.43 (95% CI 1.02, 1.99), *p* = 0.036). We found no evidence of any associations with IQ at 8 years.

**Conclusion:**

In a population in which fish intakes were below national recommendations, our results illustrate the importance of seafood intake in children on behavioural variables, specifically prosocial behaviour. Further research on the association of seafood intake with a wider range of indicators of child neurodevelopment will provide stronger evidence of the role of seafood intake in cognitive development.

**Supplementary Information:**

The online version contains supplementary material available at 10.1007/s00394-025-03636-7.

## Introduction

Optimal neurodevelopment in childhood relies on adequate dietary intake of specific nutrients. Seafood is a rich source of many macro- and micronutrients, including protein, long-chain fatty acids, iodine, selenium, choline and vitamin D [1]. In particular, fish and shellfish are the main dietary sources of the long-chain *n*-3 polyunsaturated fatty acid (LC-PUFA) docosahexaenoic acid (DHA), as well as a predominant source of eicosahexaenoic acid (EPA) [2], which are crucial for cognitive function and development. EPA and DHA can be synthesised endogenously from alpha-linolenic precursors, but the process has limited capacity and a dietary intake is needed to maintain adequate levels [3]. Their roles span from regulating gene expression to influencing membrane fluidity, for which they are especially highly concentrated in the brain [3, 4]. Iodine is an essential micronutrient in the synthesis of thyroid hormones and mediates the effect of thyroid hormone on the brain, making it critical to neurodevelopment in children [5]. Selenium is an essential component of selenoproteins, which are involved in DNA manufacture, act as antioxidants and are critical for the metabolism of thyroid hormones. Choline is essential for the production of acetylcholine, which is involved in the pain response and in cognitive functions. Vitamin D promotes calcium absorption in the intestinal system and maintains serum calcium and phosphorus concentrations to enable normal bone mineralisation; other functions include involvement in inflammation reduction and modulation of cell growth, neuromuscular and immune functions, and glucose metabolism.

A Joint Food and Agricultural Organization/World Health Organization (FAO/WHO) Expert Consultation in 2023 noted the strong benefits of fish consumption at all life stages, including childhood, but acknowledged that risk–benefit assessments are needed at regional, national and sometimes local levels to reflect difference in local consumption, fish contamination levels and nutrient content, plus the nutritional status of the population, cultural habits and demographics [6]. In the UK it is recommended that children eat at least two portions of fish a week, including at least one portion of oily fish (girls are recommended to have no more than two portions of oily fish per week in recognition of increased vulnerability from dioxins and dioxin-like polychlorinated biphenyls (PCBs) due to future reproductive capability) [7, 8]. However, data from the National Diet and Nutrition Survey (NDNS) (2011–2018) suggest that the mean intake of total seafood is well below this recommendation in children [9], and this may therefore restrict the intake of critical nutrients such as LC-PUFA and iodine. However, in contrast, seafood is also a source of exposure to mercury, which is a neurotoxin and may adversely affect neurodevelopment [10, 11], as well as other toxins such as dioxins and PCBs. The tension between these conflicting factors has made the development of public health messages on fish consumption in children problematic and may contribute to the low intakes found in children.

Previous studies on seafood and neurodevelopment have focused mainly on the possible benefits of maternal seafood intake during pregnancy on the offspring’s neurodevelopment rather than the child’s intake. For example, a systematic review of studies on maternal seafood intake and child neurodevelopment published from 2000 to 2019 identified 26 articles (from 18 prospective studies) [12], whereas a similar systematic review of child seafood intakes and neurodevelopment, also of studies published from 2000 to 2019, identified only 13 articles (six randomised controlled trials plus seven articles from six prospective studies) [13]. The overall conclusion from the latter systematic review on child seafood intakes was that there was an inadequate number of studies available, and therefore insufficient evidence to determine associations with measures of developmental domains, academic performance, autism spectrum disorder, attention deficit disorder or anxiety/depression.

Data from the longitudinal cohort study the Avon Longitudinal Study of Parents and Children (ALSPAC) has been used to model associations between maternal seafood intake during pregnancy and a range of cognitive and developmental outcomes in the offspring [14–17]. In general, these studies have shown no detrimental associations of maternal seafood intake with child outcomes, and in some cases, a positive association, suggesting that the adverse effects of mercury may be outweighed by the beneficial effects of nutrients at the level of seafood intake, and mercury exposure, in the cohort [18]. However, there has been less focus on the seafood intake of the child and its associations with neurocognitive development: such studies could make a valuable contribution to addressing the insufficiency of evidence identified in the systematic review [13].

Our aim, therefore, was to use data from ALSPAC to model associations between seafood intake in childhood (at age 7 years) and cognitive development (intelligence quotient (IQ)) at age 8 years and behavioural development (Strength and Difficulties Questionnaire (SDQ) scores) at ages 7 and 9 years. Addressing this evidence gap will ultimately contribute to guiding the content and implementation of public health nutrition messages on fish eating in childhood.

## Methods

### ALSPAC/the participants

ALSPAC is a large prospective observational study, established to explore environmental and genetic factors affecting a person’s health and development. All pregnant women resident in Avon, UK with expected dates of delivery between 1 April 1991 and 31 December 1992 were invited to take part in the study. Of the 20,248 pregnancies identified as being eligible, the number enrolled was 14,541 resulting in 14,062 live births and 13,988 children alive at 1 year of age [19, 20]. When the oldest children were approximately 7 years of age, the initial sample was bolstered with eligible cases who had failed to join the study originally, resulting in an additional 913 children being enrolled.

Further details regarding ALSPAC are available at https://www.bristol.ac.uk/alspac/. The study website contains details of all the data that are available through a fully searchable data dictionary and variable search tool which can be found at http://www.bristol.ac.uk/alspac/researchers/our-data/.

### Exposures: dietary data

ALSPAC collected dietary data about enrolled children when they were 7 years old, using a postal food frequency questionnaire (FFQ, completed by the primary caregiver). These data were selected as they are chronologically prior to the outcome measure and contain a reliable measure of fish intake in a large number of participants. The questionnaire included questions about the frequency and habitual consumption of 80 food and drink types [21]. Caregivers were asked to select how often their child ate these foods or drinks from five options: never/rarely, once in two weeks, one to three times a week, four to seven times a week, more than once a day. There were five questions about seafood intake: shellfish, white fish in breadcrumbs or batter, white fish without coating, tuna, other fish (pilchards, sardines, mackerel, herring, kippers, trout, salmon, etc.). The food’s nutritional content was obtained from UK food tables [22]. The total weight of seafood per week (g/week) was calculated by summing the frequency of intake multiplied by a standard portion size for each type of seafood for 7-year-olds (about 95 g) [23]. For white fish coated in breadcrumbs or batter, a value of 60 g fish/100 g product was used to calculate total seafood intake. Estimates of DHA intake were based on values from food composition tables: white fish 0.32 g/100 g, oily fish 0.89 g/100 g, shellfish 0.34 g/100 g [24].

The use of FFQs to estimate seafood intake in ALSPAC has been validated previously [14]. The complete questionnaire is available online through the ALSPAC website (My Son/Daughter at School) [25].

### Outcomes: IQ and SDQ scores

The outcome measures used in this study were: (1) IQ scores at 8 years (Verbal, Performance and Total); (2) SDQ scores at 7 and 9 years (Total difficulties, Hyperactivity, Emotion difficulties, Conduct difficulties, Peer difficulties, Prosocial strengths).

IQ was assessed in ALSPAC using a short form of the internationally recognised Weschler Intelligence Scale for Children (WISC-III UK) [26, 27]. At age 8 years, children were invited to attend a clinic to assess cognitive function. The test comprised five verbal and five performance subtests and the forward and backwards digit span task. These produced an individual score and were combined/scaled to produce a total score (scores were prorated according to WISC instructions to generate a total score for participants who completed only four of the five tasks, with the exception of substitution of the digit span score in place of an unobtained fifth verbal subtest). Trained psychologists conducted all testing, and a senior psychologist ensured inter-rater reliability by supervising testers and checking scoring [14]. For this study, we defined suboptimal cognitive outcomes as scores in the lowest quartile of the distribution for verbal, performance and total IQ, an identical categorisation to that in the analysis by Hibbeln et al. [14] of maternal prenatal seafood intake and offspring cognitive outcomes in ALSPAC.

The parent-completed version of the SDQ was used in questionnaires at age 7 and 9 years [28–30]. The responses were used to create five individual component scores and an overall behaviour score. The details of the derivation are contained in Goodman [28]. The scores were categorised into four groups: Close to average/Slightly raised/High/Very high (for Prosocial the categories were defined as Close to average/Slightly lowered/Low/Very low) (see Supplementary Table 1) [31].

### Confounders

Possible confounding factors were identified from previous papers and academic consideration of potential associations [14, 17, 32]. The data were collected by ALSPAC using questionnaires completed by the mothers. The following categorical factors were included as part of the adjusted regression models: child sex; maternal education, based on the highest educational qualification in the UK examination system (Low; None/CSE/vocational; Medium – O-level; or High – A-level/degree); maternal smoking status in the first trimester of pregnancy (yes or no); maternal alcohol consumption in the first trimester of pregnancy (yes or no); maternal seafood consumption in pregnancy, divided into the three categories to represent intakes compliant or not with the UK recommendations (0, 1–340 or ≥ 340 g/week) as used by Hibbeln et al. [14]; parity of mother (0, 1, ≥ 2); Family Adversity Index (FAI) (No adversities, Few adversities, Many adversities) (see Supplementary Text for details); preterm delivery (yes or no); birthweight (low, normal/high); child ethnicity (white or non-white) and breastfeeding (ever or never). The following continuous variables were included: maternal age; child’s age at the time of IQ or SDQ testing (months).

### Statistical analyses

Statistical analysis was performed using SPSS version 29.0.1.0 (IBM SPSS Statistics). Participants were excluded from the final analysis if they had missing data on seafood consumption or IQ or SDQ and confounders in the case of adjusted analyses (all available cases analysis).

To conduct analyses, three categories of seafood consumption were derived: none, consumption of 1–190 g/week (the equivalent of up to two portions per week) and consumption of ≥ 190 g/week (the equivalent of two or more portions a week). These were based on previously age-adjusted portion sizes for ALSPAC children (7-year-old child’s portion of seafood is about 95 g (equivalent to a recommendation of at least 190 g fish/week)) [33]. Suboptimal IQ was defined as the lowest quartile of scores for IQ, with scores in the three highest quartiles combined being optimal. Suboptimal SDQ scores were defined as High/Very high, with Close to average/Slightly raised being optimal (for Prosocial the categories were defined as Low/Very low, with Close to average/Slightly lowered being optimal).

For summary statistics, continuous values are reported as mean ± standard deviation (SD) and 95% confidence intervals (CI); chi-square tests were used to analyse differences in categorical data and ANOVA for continuous data. The demographics of the participants were analysed as follows for IQ at 8 years, and SDQ at 7 and 9 years: (1) demographics of included participants; (2) demographics of those with valid dietary and IQ data or SDQ scores at 7 or 9 years versus those without; (3) demographics of included participants by seafood consumption category. The mean IQ and SDQ scores for each category of seafood intake were also calculated.

Logistic regression was used to examine the effect of the seafood intake category on being in the suboptimal IQ group or suboptimal SDQ score group compared with the reference categories. The reference category for seafood intake was ≥ 190 g/week. The regression analyses were adjusted for sex, age at testing, maternal variables (maternal education, maternal age, maternal smoking status in pregnancy, maternal alcohol consumption in pregnancy and parity), FAI, preterm, birthweight, child ethnicity and breastfeeding. The linear trend across seafood intake categories was assessed for all regression analyses by including the derived childhood seafood intake variable as a continuous variable in the regression model. In all models, the correlation coefficient for maternal versus child seafood intake were < 0.20 so the assumptions of regression were not violated.

The adjusted logistic model was repeated for DHA intake from fish as a percentage of energy intake for those outcome variables for which there were associations for seafood.

Nagelkerke R-squared values were used to test the model fit.

## Results

**Fig. 1:**
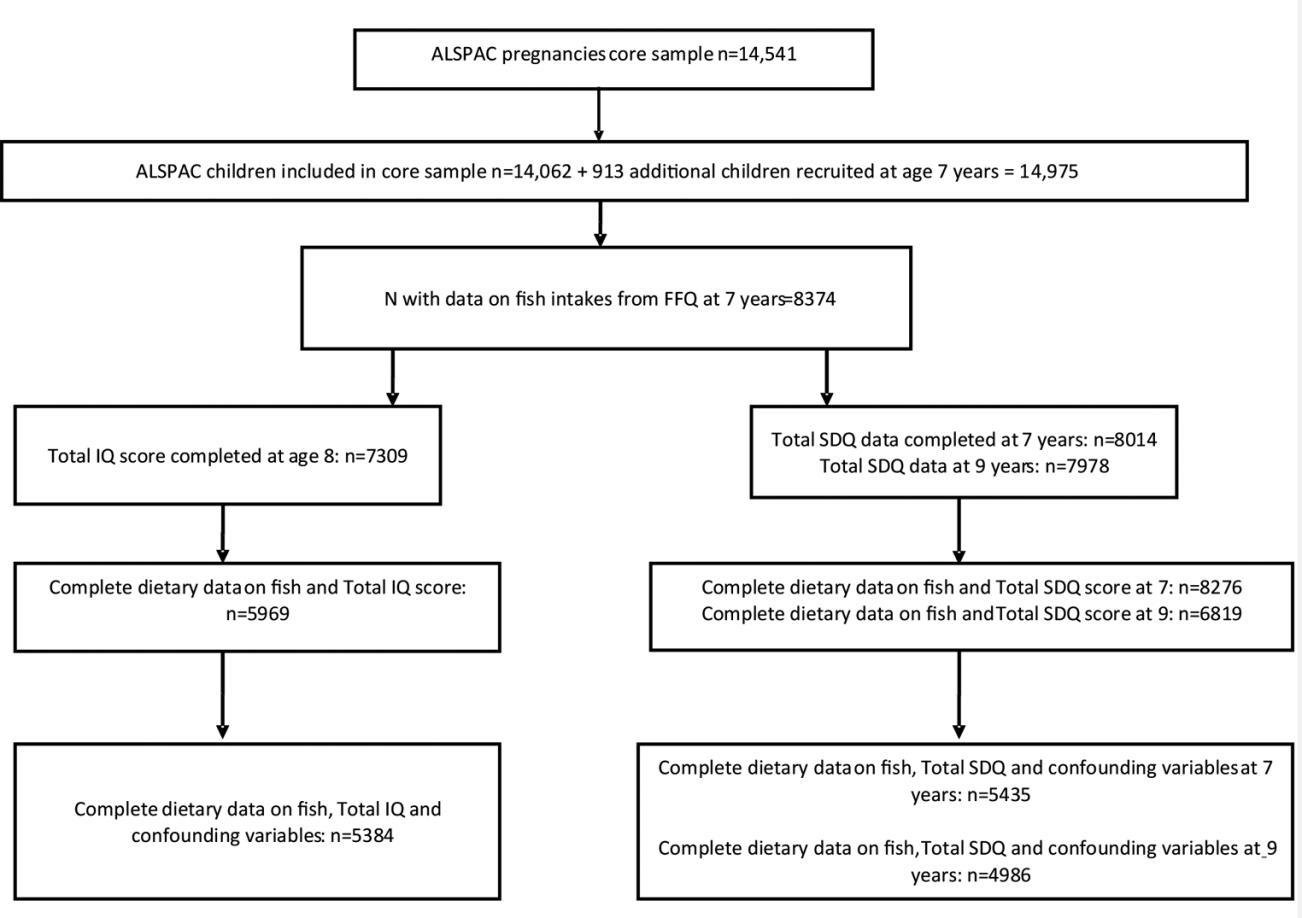
Study flow diagram for participant data from the Avon Longitudinal Study of Parents and Children (ALSPAC)

9006 participants from the original sample were excluded due to missing dietary and/or IQ data; 6699 were excluded for missing SDQ at 7 years; and 8156 were excluded for missing SDQ at 9 years. This left the final study sample as 5969 children with complete dietary and IQ data, 8276 with complete dietary data and SDQ score at 7 years, and 6819 with complete dietary data and SDQ scores at 9 years (Fig. 1). Table 1 shows the demographics of the included participants for each of the three scores. Supplementary Table 2 shows the demographics of those with complete dietary and IQ/SDQ data compared with those without. The included participants were generally from a more affluent demographic (more likely to own or have a mortgaged house, higher social class and higher maternal education level). Mothers of the included children were more likely to have consumed some alcohol in pregnancy than none and were more likely not to have smoked when pregnant. There was no difference in the distribution of childhood seafood intake categories between children with dietary and IQ/SDQ data and children without. For the maternal seafood intake category, the included participants’ mothers were more likely to be in the highest category (≥ 340 g) and less likely to be in the 0 g category of prenatal seafood intake. Supplementary Table 3 shows the demographic characteristics across categories of seafood intake in childhood. The median childhood seafood consumption was 123 (IQR 61–202) g/week. 7.2% of children ate 0 g/week, 63.9% ate 1–190 g/week, and 28.9% ate ≥ 190 g/week. White-coated fish products comprised almost half the mean total seafood intake (46%), while oily fish comprised only 7% by weight.

**Table 1:** Demographics of included ALSPAC participants (complete data on fish intake at 7 years and IQ score at 8 years, Strengths and Difficulties Questionnaire (SDQ) score at 7 years, or SDQ score at 9 years)

Characteristic	IQ		SDQ			
	8 years		7 years		9 years	
	*n*	%	*n*	%	*n*	%
**Childhood seafood intake at 7 years (g/week)**						
0	417	7.0	590	7.1	475	7.0
1-190	3824	64.1	5292	63.9	4376	64.2
≥190	1728	28.9	2394	28.9	1968	28.9
**Maternal education status**						
Low (None/CSE/Vocational)	1175	20.0	1908	23.7	1429	21.4
Medium (O level)	2041	34.8	2836	35.2	2337	35.0
High (A level/Degree)	2645	45.1	3312	41.1	2949	43.7
**Maternal age at delivery years)**						
≤20	143	24.0	298	3.6	195	2.9
21–30	3530	59.1	5031	60.8	4064	59.6
≥31	2295	38.5	2946	35.6	2560	37.5
**Maternal seafood intake in pregnancy g/week)**						
0	637	11.0	930	11.8	723	11.0
1-340	3639	63.3	4989	63.2	4151	63.3
≥340	1475	25.6	1972	25.0	1681	25.6
**Maternal smoking status during pregnancy**						
No	4887	83.1	6518	80.2	5556	82.7
Yes	996	16.9	1605	19.8	1160	17.3
**Maternal alcohol consumption during pregnancy**						
No	2574	43.8	3580	44.1	2942	43.9
Yes	3305	56.2	4531	55.9	3761	56.1
**Child’s sex**						
Male	2976	49.9	4241	51.3	3455	50.7
Female	2992	50.1	4023	48.7	3356	49.3
**Child’s ethnicity**						
White	5749	93.3	7902	98.3	6566	98.4
Other than white	93	1.6	140	1.7	104	1.6
**Parity**						
0	2729	47.4	3666	46.2	3087	47.0
1	2065	35.9	2823	35.6	2344	35.7
2+	961	16.7	1448	18.2	1136	17.3
**Breastfed**						
Yes	4663	80.4	6092	77.4	5192	79.0
No	1093	19.0	1774	22.6	1379	21.0
**Family Adversity Index**						
No adversity	2583	43.3	3390	41.0	2881	43.2
Few adversities	2578	43.2	3646	44.1	2969	43.8
Many adversities	807	13.5	1239	15.0	952	14.0
**Birthweight**						
Low (< 2500 g)	262	4.4	361	4.4	280	4.2
Normal (≥ 2500)	5630	95.6	7816	95.6	6451	95.8
**Gestation**						
Preterm (< 37 weeks)	285	7.7	425	5.1	326	4.8
Term (≥ 37 weeks)	3432	92.3	7850	94.9	6493	95.2

Table 2 shows the mean IQ and SDS scores by seafood intake category. There were no associations in mean IQ between categories. Higher fish intake was associated with a higher Prosocial score, and lower Hyperactivity, Conduct, Peer problems and Total difficulties scores at 7 years old. At 9 years old, these associations were still evident for Prosocial, Peer Problems and Total difficulties scores.

**Table 2:** Mean IQ scores at 8 years and Strengths and Difficulties Questionnaire (SDQ) scores at 7 and 9 years by seafood intake at 7 years in ALSPAC

	Child seafood intake (g/week)			*P* value	*n*
	0	1-190	≥ 190		
Cognition					
IQ at 8 years					
Verbal	109.4 ± 16.7 (108.0-111.0)	107.8 ± 16.7 (107.3-108.4)	108.0 ± 16.7 (107.2-108.8)	0.194	5997
Performance	100.4 ± 16.5 (98.8-102.1)	100.2 ± 17.0 (99.7-100.8)	100.6 ± 16.9 (99.8-101.4)	0.725	5986
Total	106.0 ± 16.3 (104.5-107.6)	104.9 ± 16.4 (104.4-105.5)	105.2 ± 16.2 (104.4–106.0)	0.379	5969
Behaviour					
SDQ subscores at 7 years					
Prosocial	7.92 ± 1.93 (7.77, 8.08)	8.12 ± 1.75 (8.07, 8.17)	8.35 ± 1.69 (8.28, 8.42)	< 0.001	8326
Hyperactivity	3.41 ± 2.49 (3.20, 3.61)	3.45 ± 2.37 (3.38, 3.51)	3.26 ± 2.34 (3.16, 3.35)	0.005	8310
Emotional	1.61 ± 1.71 (1.47, 1.75)	1.51 ± 1.68 (1.46, 1.55)	1.47 ± 1.63 (1.41, 1.54)	0.209	8318
Conduct	1.74 ± 1.54 (1.62, 1.87)	1.61 ± 1.47 (1.57, 1.65)	1.56 ± 1.45 (1.50, 1.62)	0.024	8329
Peer problems	1.23 ± 1.49 (1.11, 1.35)	1.07 ± 1.43 (1.03, 1.10)	1.00 ± 1.39 (0.95. 1.06)	0.002	8322
Total difficulties score	7.98 ± 5.14 (7.57, 8.40)	7.61 ± 4.86 (7.48, 7.74)	7.28 ± 4.74 (7.09, 7.47)	0.001	8277
SDQ subscores at 9 years					
Prosocial	8.16 ± 1.77 (8.00, 8.32)	8.32 ± 1.65 (8.23, 8.33)	8.47 ± 1.59 (8.40, 8.54)	< 0.001	6843
Hyperactivity	2.97 ± 2.38 (2.76, 3.19)	2.93 ± 2.25 (2.86, 3.00)	2.81 ± 2.16 (2.71, 2.90)	0.091	6840
Emotional	1.63 ± 1.80 (1.47, 1.80)	1.48 ± 1.72 (1.43, 1.53)	1.49 ± 1.74 (1.45, 1.53)	0.144	6829
Conduct	1.29 ± 1.40 (1.16, 1.41)	1.27 ± 1.43 (1.23, 1.32)	1.21 ± 1.27 (1.16, 1.27)	0.261	6839
Peer problems	1.31 ± 1.63 (1.16, 1.45)	1.11 ± 1.50 (1.07, 1.16)	1.01 ± 1.42 (0.95, 1.07)	< 0.001	6829
Total difficulties score	7.18 ± 5.11 (6.72, 7.64)	6.77 ± 4.90 (6.62, 6.91)	6.45 ± 4.69 (6.24, 6.65)	0.004	6819

Analysis of variance (ANOVA). Values are mean ± SD (95% CI)

Table 3 shows adjusted results for being in the suboptimal IQ or SDQ group compared with the optimal IQ and SDQ group. We found associations for the Prosocial subgroup of the SDQ at both age 7 and 9 years with seafood intake such that an intake of 0 g/week increased the odds of a suboptimal score by 35% compared with ≥ 190 g/week, and an intake of 1–190 g/week increased the odds by 25% compared with ≥ 190 g/week at age 7 years. The association was replicated at age 9 years: the odds were increased by 43% and 30%, respectively. The p for the trends at both ages suggested that the associations were linear according to seafood intake. When the associations for the Prosocial score were modelled with DHA from fish as the exposure variable, there was evidence for a weak association, with an increase of 25% in the odds of suboptimal SDQ score at 9 years (Table 4). There were no associations for the other SDQ subgroups. We found no associations between childhood seafood intake and Verbal, Performance or Total IQ. The unadjusted results are shown in Supplementary Table 4.

**Table 3:** Child seafood consumption at age 7 years and suboptimal child outcomes (IQ at age 8 years and Strengths and Difficulties Questionnaire (SDQ) scores at age 7 and 9 years in ALSPAC: adjusted logistic regression models

Outcomes	*n* (%) of children in suboptimal IQ or SDQ			Odds of suboptimal IQ or SDQ compared with the reference category (adjusted)						
	Child seafood intake (g/week)			0 vs. ≥ 190 (ref) g/week		1-190 vs. ≥ 190 (ref) g/week		*P* trend*	*n*	Nagelkerke *R*^2^
	0	1-190	≥ 190	OR (95% CI)	*P*	OR (95% CI)	*P*			
Cognition										
IQ at 7 years										
Verbal	84 (20.1%)	864 (22.5%)	382 (22.0%)	0.74 (0.54, 1.00)	0.050	0.97 (0.83, 1.13)	0.678	0.138	5412	0.126
Performance	91 (21.7%)	872 (22.7%)	380 (22.0%)	0.92 (0.69, 1.23)	0.565	1.00 (0.86, 1.16)	0.968	0.699	5399	0.073
Total	85 (20.4%)	859 (22.5%)	382 (22.1%)	0.77 (0.57, 1.04)	0.087	0.93 (0.80, 1.09)	0.933	0.110	5384	
Behaviour										
SDQ at 7 years										
Prosocial strengths	145 (24.5%)	1049 (19.7%)	397 (16.5%)	1.35 (1.01, 1.81)	0.042	1.25 (1.06, 1.47)	0.008	0.006	5454	0.042
Hyperactivity difficulties	46 (7.8%)	347 (6.5%)	142 (5.9%)	1.20 (0.76, 1.89)	0.439	1.01 (0.77, 1.31)	0.953	0.586	5456	0.055
Emotional difficulties	49 (8.3%)	349 (6.6%)	149 (6.2%)	1.32 (0.83, 2.08)	0.240	1.11 (0.86, 1.43)	0.426	0.696	5454	0.018
Conduct difficulties	78 (13.2%)	538 (10.1%)	251 (10.4%)	1.04 (0.72, 1.51)	0.842	0.86 (0.70, 1.05)	0.842	0.499	5456	0.049
Peer problems difficulties	59 (10.0%)	356 (6.7%)	146 (6.1%)	1.37 (0.88, 2.21)	0.163	1.07 (0.83, 1.39)	0.608	0.232	5452	0.034
Total difficulties score	45 (7.6%)	295 (5.6%)	123 (5.1%)	1.10 (0.66, 1.84)	0.712	0.99 (0.74, 1.32)	0.924	0.845	5435	0.065
SDQ at 9 years										
Prosocial strengths	84 (17.5%)	695 (15.8%)	249 (12.6%)	1.43 (1.02, 1.99)	0.036	1.30 (1.08, 1.57)	0.006	0.005	4998	0.051
Hyperactivity difficulties	52 (10.9%	344 (7.9%)	136 (6.9%)	1.72 (1.00, 2.95)	0.051	1.18 (0.84, 1.66)	0.337	0.073	4999	0.078
Emotional difficulties	30 (6.3%)	199 (4.5%)	66 (3.3%)	1.02 (0.64, 1.63)	0.925	0.83 (0.64, 1.07)	0.156	0.470	4989	0.034
Conduct difficulties	46 (9.6%)	277 (6.3%)	153 (7.8%)	0.94 (0.55, 1.56)	0.809	1.29 (0.98, 1.70)	0.069	0.388	4991	0.056
Peer problems	31 (6.4%)	336 (7.7%)	124 (6.3%)	1.57 (1.03, 2.40)	0.036	1.22 (0.94, 1.57)	0.138	0.032	4987	0.044
Total difficulties score	28 (5.9%)	211 (4.8%)	77 (3.9%)	1.45 (0.81, 2.59)	0.215	1.25 (0.88, 1.76)	0.213	0.147	4986	0.043

Reference categories (Optimal)

IQ: Three highest quartiles of IQ

SDQ: Score is Close to average/Slightly raised or lowered

*A test for trend was made with the assumption that the three seafood intake categories were equally spaced

Model adjusted for child sex and age at IQ testing, maternal age, maternal education, maternal prenatal smoking status and maternal prenatal alcohol consumption

Family adversity index, gestation, birthweight, child ethnic group and breastfeeding duration, maternal fish intake in pregnancy

**Table 4:** Energy-adjusted child DHA consumption from fish at age 7 years and suboptimal child outcomes (Strengths and Difficulties Questionnaire (SDQ) scores at age 7 and 9 years in ALSPAC: adjusted logistic regression models

	Odds of suboptimal SDQ subscore compared with the reference category (adjusted)								
	Quartiles 1 vs. 4 (ref) DHA %E		Quartiles 2 vs. 4 (ref) DHA %E		Quartiles 3 vs. 4 (ref) DHA %E		*P* trend	*n*	Nagelkerke *R*^2^
	OR (95% CI)	*P*	OR (95% CI)	*P*	OR (95% CI)	*P*			
Behaviour									
SDQ 7 years									
Prosocial strengths	1.16 (0.95, 1.42)	0.147	1.16 (0.95, 1.42)	0.134	1.02 (0.83, 1.25)	0.839	0.077	5380	0.04
SDQ at 9 years									
Prosocial strengths	1.25 (0.99, 1.58)	0.058	1.18 (0.94, 1.48)	0.162	1.11 (0.88, 1.40)	0.375	0.051	4929	0.05

Reference categories (Optimal): SDQ: Score Close to average/Slightly lowered

Models adjusted for child sex and age at IQ testing, maternal age, maternal education, maternal prenatal smoking status and maternal prenatal alcohol consumption, maternal fish intake, family adversity index, gestation, birthweight, child ethnic group and breastfeeding duration

DHA %E, docosahexaenoic acid as a percentage of energy

All Nagelkerke R-square values were between 0 and 1 indicating acceptable goodness of fit in the models.

## Discussion

Previous research has highlighted the importance of seafood consumption in providing essential nutrients for neurodevelopment prenatally, but there has been less research on the role of seafood intakes in childhood. We therefore investigated the relationship between childhood seafood intake at age 7 years and IQ scores at age 8 years, and SDQ scores at 7 and 9 years, using data from the ALSPAC study. We found a positive association between seafood intake and the Prosocial strengths score, a subscale of the SDQ, at both 7 and 9 years old. Our findings suggested that DHA was positively associated with the Prosocial strengths score (*n*-3 fatty acid intake during childhood is thought to have a beneficial effect on neurodevelopmental outcomes [34]). We found no evidence of associations with other subscales of the SDQ or IQ scores at age 8 years and childhood seafood intake.

A systemic review in 2019 included 15 studies (published up to 2019) describing seafood consumption in children and adolescents and neurocognitive outcomes, of which 13 reported beneficial neurocognitive outcomes associated with seafood consumption despite higher mercury exposures [35]. A moderate risk of bias and moderate generalisability but strong consistency and impact were noted. A further systematic review in 2020 of 13 papers (published from 2000 to 2019) found generally positive or null associations between childhood seafood consumption and developmental domains, specifically cognitive development, language and communication development, movement and physical development, and social-emotional and behavioural development [13]. Noting that it was stated that there was insufficient evidence in the systematic review to assess the relationship in children sufficiently, we have added a high-quality longitudinal study to the body of literature including a rigorous measure of fish consumption from an FFQ and standardised and widely used measures of developmental domains (cognitive development (IQ) and social-emotional development (SDQ)) in a large group of children enrolled in an observational cohort in the UK. Observational studies have generally identified a positive association between childhood/adolescent seafood consumption and improved behavioural or cognitive development in adjusted analyses [36–40]. As in the present study, these studies also used FFQ to collect seafood intake data, but included a broader range of outcome measures than our study, such as school grades, academic/vocabulary tests, and behavioural-focused measures such as the Nutrimenthe Neuropsychological Battery (NNB). Four studies focused on adolescents (12–18 years old) [36–38, 40], and two on children the same age as in our study (7–9 years old) [39, 40]. One of two studies found that the relationship between seafood consumption and school performance became negative at the highest seafood consumption levels [40]. They suggested this was due to greater exposure to pollutants found in seafood such as mercury. However, these studies are limited by a lack of information on seafood portion sizes and sub-types of seafood due to limited dietary data collection.

Randomised controlled trials (RCTs) have generally also found a positive association between seafood consumption and neurodevelopmental markers, although several factors limit the strength of evidence [41–44]. These RCTs compared various neurocognitive outcomes for children receiving seafood meals versus meat meals for a range of ages (4–6 [41, 42], 8–9 [43], 14–15 [44] years old). Two studies identified an increase in overall cognitive performance scores [41, 43] and two noted an increase in sub-scores: processing speed [44] and symbol search/picture concepts [42]. The authors of the latter study suggested that seafood intake may benefit some cognitive domains (i.e. symbol search/picture concepts, evaluating non-verbal fluid intelligence) without influencing global IQ development [42]. In general, the findings from these RCTs have to be interpreted with caution due to potentially low adherence to dietary interventions and the relatively short duration of the intervention (typically 2–3 months), but together they suggest a potential role for seafood, possibly through the action of *n*-3 LC-PUFA, which are essential as brain membrane phospholipids [45]).

We found a strong and temporally consistent association between the Prosocial strengths subscore of the SDQ and seafood consumption in childhood. The Prosocial subscore represents the child’s ability to carry out actions intended to benefit one or more people other than him/herself (subdivided as helping/sharing/comforting [46]). This ability is thought to emerge between the first and second birthday and increases in frequency and complexity as the child grows older, but with individual variability [46]. It benefits the individual, the group and wider society. Studies using the SDQ in childhood have generally focused on mercury exposure or interventions of fatty fish or fish oil rather than total fish: for example, Sarzo et al. [47] found no association between cord total mercury and SDQ measures at 7 and 11 years, with the beneficial effects of the nutrients thought to offset the neurotoxic effects of mercury; an RCT with an intervention of fatty fish rather than total fish in 4–6-year-old children found no difference in the SDQ scores after 16 weeks (FINS-KIDS Study) [48]; another study with an intervention of fish oil for 6 weeks to children aged 7–12 years old with disruptive behaviour disorders found that the intervention was associated with worsening of the Conduct subscale but a reduction in Hyperactivity [49].

We did not find an association between childhood seafood intake and IQ. Several factors may account for the inconsistencies between our findings and those of previous studies on child seafood/fish intakes and IQ. It is possible we did not find an association because our study’s seafood intake did not align with a specific phase of neurodevelopment [4]. Most of the pre-existing literature identifying a positive association between seafood intake and neurodevelopmental outcomes has focused on the adolescent period (10–16 years) or earlier childhood (0–6 years). Other factors (such as school quality, sibling order or diet quality) may be more significant in determining IQ at this time. This is consistent with a meta-analysis (2014) that proposed a critical period of brain development in infancy where LC-PUFA intake plays a more significant role [50] (for example, it is known that breast-feeding has a positive association with IQ in the ALSPAC cohort [51]). We studied the effect of diet at 7 years, but it would also be of interest to model the effect of earlier childhood fish intakes on the neurodevelopmental outcomes to help to identify the range of the critical window more clearly. In addition, it is possible that the IQ test is not sensitive enough to reflect small changes in cognition, and/or the seafood intakes may be too low or not have enough variance to detect an effect. A positive association between childhood seafood intake and IQ may also have been counterbalanced by the adverse effects of mercury and other toxins, also found in seafood. Previous research has highlighted the detrimental effects of prenatal mercury exposure on child development [52], although other studies have found the prenatal fish exposure is beneficial to neurodevelopment despite its mercury content [18]. Since blood mercury levels and seafood intake were collinear, our model did not adjust for mercury levels. Further research on diet and neurodevelopment would be needed to elucidate if mercury played a role in these results.

Our results also need to be considered in the light of seafood intakes in relation to national recommendations. We included all children with available data enrolled in ALSPAC, rather than excluding those with specific health conditions, in order for our results to be interpreted in the context of population guidance in fish intakes for children. Current UK guidance, based on a 2004 report published by the Scientific Advisory Committee on Nutrition (SACN), recommends that children eat at least two portions of fish a week, including at least one portion of oily fish (no more than two portions of oily fish per week for girls) [7, 8]. A 7-year-old child’s portion of seafood is about 95 g (recommendation equivalent to at least 190 g fish/week) [33]. Data from the UK National Diet and Nutrition Survey (NDNS) (2011–2018) suggest that the mean intake of total seafood is well below this across all age groups, particularly in children [1]: 4–18-year-olds ate less than 20 g oily fish per week, rather than the recommended minimum amount of about 95 g [9]. Possible factors contributing to this may be a taste/smell preference, family preference, lack of access/availability and cost [53]. Previous guidance around appropriate seafood consumption may also be linked to this underconsumption due to risk aversion concerning toxins in fish [54], particularly around methylmercury exposure [52]. There is a need for increased public awareness and better implementation of the recommendations. This was endorsed by the FAO/WHO Expert Consultation in which it was concluded that fish provides energy, protein and a range of other nutrients that are important for health [6].

There are several strengths to our study. Using ALSPAC data (a large population from a geographically defined area with long-term follow-up) allowed us to compare the IQs of large numbers of children with dietary data and adjust for various social/demographic factors [32]. The large study size gave our analyses greater statistical power than existing studies. Our study also used FFQs to collect dietary data, which offer reliable information about habitual dietary patterns. Alternative collection methods, such as food diaries, risk not recording less frequently consumed food and drink items or over-representing them. ALSPAC-specific FFQs have also been validated for estimating seafood intake in ALSPAC participants, increasing our confidence in these results [55].

There are also some limitations. NHS guidelines recommend at least two portions of seafood a week [7], equivalent to 190 g for 7-year-olds [33]. The seafood intake in the present study was comparatively low (median intake 123 g/week). Hence, while the data are broadly representative of UK seafood eating habits, it limits our ability to compare cognitive outcomes across the entire recommended seafood intake range. In addition, the study’s attrition rate is higher for those with evidence of social disadvantage. The cohort is also predominantly white, which may limit the generalisability of our results to the broader population. Third, as with any observational study, it is possible we did not include all potential confounding factors. Our final model explained 13.4% of the variance in verbal IQ, 7.9% in performance IQ and 12.5% in total IQ, but there may be other important variables that we were not able to include. Finally, the IQ measure was an alternate item strategy instead of the full WISC format. However, this alternate form has been previously validated compared with the full-item WISC IQ system [17].

Some studies have focused on a broader range of outcome measures than our study, but we were able to include analyses on IQ and a widely used developmental score (SDQ) at two time points. An analysis with a more expansive range of outcome measures, such as the Denver Developmental Screening Test, or other IQ measures, would provide further evidence [14].

Finally, the discrepancy between our findings and the anticipated results could be attributed to our study’s limited variability in seafood intake. Our study assessed 5885 children, of which the majority consumed 1–190 g seafood/week, and the median intake was 123 g/week. The absence of an association in our study may be due to the low variability in seafood intake, particularly the small number of children in the 0 g seafood/week group. The WISC IQ test, and possibly the SDQ, as used in this study may not have been sensitive enough to detect small changes associated with the low variability in seafood intake.

## Conclusion

In conclusion, we found evidence of an association of low seafood intake at age 7 years with a suboptimal score on the Prosocial strengths scale of the SDQ at age 7 and 9 years. There was no evidence that not eating seafood at age 7 years was associated with a suboptimal IQ at age 8 years. In a population in which fish intakes were below national recommendations, this study illustrates the important effect of seafood intake in children on behavioural variables. Seafood is a source of many vital nutrients essential for child development, so a lower-than-recommended seafood intake could still adversely affect overall health [54]. Further research on the association of seafood intake with a broader range of indicators of child neurodevelopment would provide stronger evidence of the role of seafood consumption in cognitive development, which would inform recommendations and public health guidance.

## Electronic supplementary material

Below is the link to the electronic supplementary material.

Supplementary Material 1

## Data Availability

Data are available to researchers on application to the ALSPAC Executive Committee.
