# Supplementary material for: Seafood intake in children at age 7 years and neurodevelopmental outcomes in an observational cohort study (ALSPAC)
Source: Eur J Nutr. 2025 Mar 11;64(3):120. doi: 10.1007/s00394-025-03636-7 (PMC11893685; doi:10.1007/s00394-025-03636-7)
Supplement: Supplementary file 1 — Supplementary Material 1 [file 394_2025_3636_MOESM1_ESM.docx]

**Supplementary Text**

FAI is an ALSPAC scoring index, based on Rutter’s indicators of adversity^4^. We included the short index, measured at 2–4 years old, as this was measured closest in time to our dietary and IQ/SDQ data. It consists of 15 items covering areas including family, finances, crime and education. Where more than half the items are valid, scores are calculated by giving a score of 1 for present items or 0 to non-present items. Scores range from 0 to 15 with a higher score being considered indicative of a higher level of adversity. Since the data distribution as heavily skewed, they were categorised into: 0, no adversity; 1–2, few adversities; ≥3, many adversities^5^.

**Supplementary tables**

**Supplementary Table 1**SDQ cut-off scores and banding for age 4$-$18 years

|  | **Close to average** | **Raised** | **High** | **Very high** |
| --- | --- | --- | --- | --- |
| Parent-completed SDQ score |  |  |  |  |
| Total difficulties | 0–13 | 14–16 | 17–19 | 20–40 |
| Emotional difficulties | 0–3 | 4 | 5–6 | 7–10 |
| Conduct difficulties | 0–2 | 3 | 4–5 | 6–10 |
| Hyperactivity difficulties | 0–5 | 6–7 | 8 | 9–10 |
| Peer difficulties | 0–2 | 3 | 4 | 5–10 |
| Impact difficulties | 0 | 1 | 2 | 3–10 |
|  |  |  |  |  |
|  | **Close to average** | **Lowered** | **Low** | **Very low** |
| Prosocial strengths | 8–10 | 7 | 6 | 0–5 |

See youth*in*mind [31].

Scores were further categorised into: Close to average/Raised (Optimal), High/Very high (Suboptimal) or Close to average/Lowered (Optimal), Low/Very Low (Suboptimal)

**Supplementary Table 2** Demographics of included versus excluded ALSPAC participants (complete data on fish intake at 7 years and IQ score at 8 years, Strengths and Difficulties Questionnaire (SDQ) score at 7 Years, or SDQ score at 9 years versus those without)

| **Variable** | **IQ at 8 years** | | **SDQ at 7 years** | | **SDQ at 9 years** | |
| --- | --- | --- | --- | --- | --- | --- |
|  | **Excluded  (missing dietary and/or IQ data)** | **Included (complete dietary and IQ data)** | **Excluded  (missing dietary and/or SDQ data)** | **Included (complete dietary and SDQ data)** | **Excluded  (missing dietary and/or SDQ data)** | **Included  (complete dietary and SDQ data)** |
| **n** | 9118 (60.4%) | 5969 (39.6%) | 6811 (45.1%) | 8276 (54.9%) | 8268 (54.8%) | 6819 (45.2%) |
| **Seafood intake in childhood (g/week)** |  |  |  |  |  |  |
| 0 | 0 | 417 (%) | 11 (11.2%) | 590 (7.1%) | 126 (8.1%) | 475 (7.0%) |
| 1-190 | 0 | 3824 (64.1%) | 61 (62.2%) | 5292 (63.9%) | 977 (62.8%) | 4376 (64.2%) |
| ≥190g | 0 | 1728 (28.9%) | 26 (26.5%) | 2394 (28.9%) | 452 (29.1%) | 1968 (28.9%) |
| **Maternal education status** |  |  |  |  |  |  |
| Low (None/CSE/Vocational) | 295 (32.3%) | 1175 (20.0%) | 1839 (41.8%) | 1908 (23.7%) | 2318 (40.2%) | 1429 (21.4%) |
| Medium (O level) | 322 (35.3%) | 2041 (34.8%) | 1476 (33.6%) | 2836 (35.2%) | 1975 (34.2%) | 2337 (35.0%) |
| High (A level/Degree) | 295 (32.3%) | 2645 (45.1%) | 1082 (24.6%) | 3312 (41.1%) | 1475 (25.6%) | 2949 (43.7%) |
| **Maternal age at birth (years)** |  |  |  |  |  |  |
|  | 79 (7.5%) | 143 (24.0%) | 713 (12.3%) | 298 (3.6%) | 816 (11.3%) | 195 (2.9%) |
| ≥20–29 | 680 (64.6%) | 3530 (59.1%) | 3794 (65.7%) | 5031 (60.8%) | 4761 (65.8%) | 4064 (59.6%) |
| ≥30 | 293 (27.9%) | 2295 (38.5%) | 1269 (22.0%) | 2946 (35.6%) | 1655 (22.9%) | 2560 (37.5%) |
| **Maternal seafood intake in pregnancy (g/week)** |  |  |  |  |  |  |
| 0 | 115 (12.9%) | 637 (11.0%) | 679 (15.8%) | 930 (11.8%) | 886 (15.7%) | 723 (11.0%) |
| 1-340 | 553 (62.1%) | 3639 (63.3%) | 2706 (63.0%) | 4989 (63.2%) | 3544 (63.0%) | 4151 (63.3%) |
| ≥340 | 223 (25.0%) | 1475 (25.6%) | 907 (21.1%) | 1972 (25.0%) | 1198 (21.3%) | 1681 (25.6%) |
| **Housing status** |  |  |  |  |  |  |
| Mortgaged/owned | 708 (73.0%) | 4979 (10.9%) | 3270 (61.1%) | 3574 (81.5%) | 4262 (63.2%) | 5582 (83.7%) |
| Rented/other | 262 (27.0%) | 866 (14.8%) | 2079 (38.9%) | 1496 (18.5%) | 2487 (36.8%) | 1088 (16.3%) |
| **Maternal smoking in pregnancy** |  |  |  |  |  |  |
| No | 746 (76.4%) | 4887 (83.1%) | 3424 (67.1%) | 6518 (80.2%) | 4386 (67.4%) | 5556 (82.7%) |
| Yes | 230 (23.6%) | 996 (16.9%) | 1680 (32.9%) | 1605 (19.8%) | 2125 (32.6%) | 1160 (17.3%) |
| **Maternal alcohol consumption in pregnancy** |  |  |  |  |  |  |
| No | 445 (45.9%) | 2574 (43.8%) | 2386 (47.6%) | 3580 (44.1%) | 3024 (47.1%) | 2942 (43.9%) |
| Yes | 525 (54.1%) | 3305 (56.2%) | 2623 (52.4%) | 4531 (55.9%) | 3393 (52.9%) | 3761 (56.1%) |
| **Child’s sex** |  |  |  |  |  |  |
| Male | 523 (49.7%) | 2976 (49.9%) | 3258 (51.9%) | 4241 (51.3%) | 4044 (52.3%) | 3455 (50.7%) |
| Female | 529 (50.3%) | 2992 (50.1%) | 3024 (48.1%) | 4023 (48.7%) | 3691 (47.7%) | 3356 (49.3%) |
| **Child’s ethnicity** |  |  |  |  |  |  |
| White | 876 (96.8%) | 5749 (93.3%) | 4138 (95.8%) | 7902 (98.3%) | 5474 (96.2%) | 6566 (98.4%) |
| Other than white | 29 (3.2%) | 93 (1.6%) | 183 (4.2%) | 140 (1.7%) | 219 (3.8%) | 104 (1.6%) |
| **Parity** |  |  |  |  |  |  |
| 0 | 411 (43.5%) | 2729 (47.4%) | 2127 (43.2%) | 3666 (46.2%) | 2706 (43.0%) | 3087 (47.0%) |
| 1 | 321 (34.0%) | 2065 (35.9%) | 1662 (33.7%) | 2823 (35.6%) | 2141 (34.0%) | 2344 (35.7%) |
| 2+ | 212 (22.4%) | 961 (16.7%) | 1140 (23.1%) | 1448 (18.2%) | 1452 (23.1%) | 1136 (17.3%) |
| **Breastfed** |  |  |  |  |  |  |
| Yes | 549 (72.9%) | 4663 (80.4%) | 1978 (64.2%) | 6092 (77.4%) | 2878 (65.7%) | 5192 (79.0%) |
| No | 204 (27.1%) | 1093 (19.0%) | 1105 (35.8%) | 1774 (22.6%) | 1500 (34.3%) | 1379 (21.0%) |
| **Family Adversity Index (FAI)** |  |  |  |  |  |  |
| None (0) | 547 (52.0%) | 2583 (43.3%) | 3844 (60.5%) | 3390 (41.0%) | 4353 (55.7%) | 2881 (43.2%) |
| Few (1–2) | 382 (36.3%) | 2578 (43.2%) | 1910 (30.0%) | 3646 (44.1%) | 2570 (32.9%) | 2969 (43.8%) |
| Many (≥ 3) | 123 (11.7%) | 807 (13.5%) | 603 (9.5%) | 1239 (15.0%) | 890 (11.4%) | 952 (14.0%) |
| **Birthweight** |  |  |  |  |  |  |
| Low (< 2500g) | 75 (20.1%) | 262 (4.4%) | 450 (7.9%) | 361 (4.4%) | 531 (7.4%) | 280 (4.2%) |
| Normal (≥ 2500) | 298 (78.9%) | 5630 (95.6%) | 5243 (92.1%) | 7816 (95.6%) | 6608 (92.6%) | 6451 (95.8%) |
| **Gestation** |  |  |  |  |  |  |
| Preterm (< 37 weeks) | 86 (13.3%) | 285 (7.7%) | 471 (8.2%) | 425 (5.1%) | 570 (7.9%) | 326 (4.8%) |
| Term (≥ 37 weeks) | 559 (86.7%) | 3432 (92.3%) | 5303 (91.8%) | 7850 (94.9%) | 6660 (92.1%) | 6493 (95.2%) |

**Supplementary Table 3** Demographics of participants in ALSPAC by childhood seafood consumption at 7 years with chi-square tests of independence

| **Characteristic** | **Childhood seafood consumption at 7 years old (g/week)** | | | | | | | | | | | |
| --- | --- | --- | --- | --- | --- | --- | --- | --- | --- | --- | --- | --- |
|  | **IQ at 8 years** | | | | **SDQ at 7 years** | | | | **SDQ at 9 years** | | | |
|  | **0 (n = 417)** | **1–190 g (n = 3824)** | ≥ **190 (n = 1728)** | **P** | **0 (n = 590)** | **1–190 g (n = 5293)** | ≥ **190 (n = 2394)** | **P** | **0 (n = 475)** | **1–190 g (n = 4376)** | ≥ **190 (n = 1968)** | **P** |
| **Maternal education status** |  |  |  |  |  |  |  |  |  |  |  |  |
| Low (None/CSE/Vocational) | 97 (23.8%) | 757 (20.2%) | 321 (18.9%) | 0.058 | 166 (29.1%) | 1222 (23.7%) | 520 (22.3%) | 0.002 | 115 (24.9%) | 928 (21.6%) | 386 (20.0%) | 0.008 |
| Medium (O level) | 126 (30.9%) | 1339 (35.6%) | 576 (33.9%) |  | 178 (31.2%) | 1857 (36.0%) | 801 (34.3%) |  | 142 (30.8%) | 1544 (36.0%) | 651 (33.7%) |  |
| High (A level/Degree) | 185 (45.3%) | 1660 (44.2%) | 800 (47.1%) |  | 227 (39.8%) | 2073 (40.2%) | 1012 (43.4%) |  | 204 (44.3%) | 1820 (42.4%) | 895 (46.3%) |  |
| **Maternal age at birth (years)** |  |  |  |  |  |  |  |  |  |  |  |  |
| ≤20 | 13 (3.1%) | 104 (2.8%) | 43 (2.5%) | 0.878 | 25 (4.3%) | 210 (4.1%) | 88 (3.8%) | 0.718 | 14 (3.0%) | 145 (3.4%) | 54 (2.8%) | 0.798 |
| 21–30 | 278 (67.3%) | 2310 (61.6%) | 1070 (63.2%) |  | 352 (61.1%) | 3279 (63.5%) | 1485 (63.7%) |  | 284 (61.1%) | 2664 (62.1%) | 1212 (63.0%) |  |
| ≥31 | 122 (29.5%) | 1334 (35.6%) | 578 (34.2%) |  | 199 (34.5%) | 1676 (32.4%) | 758 (32.5%) |  | 167 (35.9%) | 1476 (34.4%) | 658 (34.2%) |  |
| **Maternal seafood intake in pregnancy (g/week)** |  |  |  |  |  |  |  |  |  |  |  |  |
| 0 | 117 (29.4%) | 414 (11.2%) | 106 (6.4%) | < 0.001 | 170 (30.2%) | 612 (12.1%) | 148 (6.5%) | < 0.001 | 137 (30.2%) | 470 (11.2%) | 116 (6.1%) | < 0.001 |
|  | 222 (55.8%) | 2479 (67.3%) | 938 (56.2%) |  | 305 (54.3%) | 3373 (66.8%) | 1311 (57.5%) |  | 248 (54.6%) | 2821 (67.0%) | 1082 (57.2%) |  |
| ≥340 | 59 (14.8%) | 791 (21.5%) | 625 (37.4%) |  | 87 (15.5%) | 1063 (12.1%) | 822 (36.0%) |  | 69 (15.2%) | 919 (21.8%) | 693 (36.6%) |  |
| **Housing status** |  |  |  |  |  |  |  |  |  |  |  |  |
| Mortgaged/owned | 318 (77.2%) | 3197 (85.4%) | 1464 (46.4%) | < 0.001 | 436 (75.3% | 4199 (81.4%) | 1939 (83.2% | 0.002 | 366 (78.5%) | 3589 (83.8%) | 1627 (84.6%) | 0.080 |
| Rented/other | 94 (22.8%) | 545 (14.6%) | 227 (53.6%) |  | 143 (24.7%) | 962 (18.6%) | 391 (16.8%) |  | 100 (21.5%) | 692 (16.2%) | 296 (15.4%) |  |
| **Household crowding (people per room at home)** |  |  |  |  |  |  |  |  |  |  |  |  |
| ≤1 | 227 (93.5%) | 3584 (96.5%) | 1619 (97.1%) | 0.002 | 537 (92.9%) | 4865 (95.5%) | 2217 (96.3%) | 0.003 | 442 (94.6%) | 4071 (96.1%) | 1842 (97.2%) | 0.022 |
|  | 27 (6.5%) | 119 (3.2%) | 48 (2.9%) |  | 41 (7.1%) | 231 (4.5%) | 84 (3.7%) |  | 25 (5.4%) | 164 (3.9%) | 53 (2.8%) |  |
| **Maternal ethnicity** |  |  |  |  |  |  |  |  |  |  |  |  |
| White | 393 (97.5%) | 3679 (98.9%) | 1654 (98.3%) | 0.150 | 552 (96.8%) | 5068 (98.5%) | 2282 (98.1%) | 0.107 | 447 (97.2%) | 4222 (98.6%) | 1897 (98.3%) | 0.044 |
| Non white | 10 ( | 42 ( | 29 ( |  | 18 (3.2%) | 77 (1.5%) | 45 (1.9%) |  | 13 (2.8%) | 58 (1.4%) | 33 (1.7%) |  |
| **Maternal smoking status during pregnancy** |  |  |  |  |  |  |  |  |  |  |  |  |
| No | 336 (81.4%) | 3113 (82.7%) | 1438 (84.4%) | 0.181 | 455 (78.6%) | 4138 (79.7%) | 1925 (81.8%) | 0.056 | 382 (81.6%) | 3456 (82.4%) | 1628 (83.8%) | 0.314 |
| Yes | 77 (18.6%) | 653 (17.3%) | 266 (15.6%) |  | 124 (21.4%) | 1054 (20.3%) | 427 (18.2%) |  | 86 (18.4%) | 759 (17.6%) | 315 (16.2%) |  |
| **Maternal alcohol consumption during pregnancy** |  |  |  |  |  |  |  |  |  |  |  |  |
| No | 181 (43.9%) | 1609 (42.8%) | 784 (45.9%) | 0.096 | 249 (43.3%) | 2253 (43.3%) | 1078 (45.9%) | 0.133 | 194 (41.6%) | 1858 (43.2%) | 890 (45.9%) | 0.090 |
| Yes | 231 (56.1%) | 2151 (57.2%) | 923 (54.1%) |  | 326 (56.7%) | 2933 (56.6%) | 1272 (54.1%) |  | 272 (58.4%) | 2439 (56.8%) | 1050 (54.1%) |  |
| **Child’s sex** |  |  |  |  |  |  |  |  |  |  |  |  |
| Male | 233 (55.9%) | 1962 (51.3%) | 781 (45.2%) | < 0.001 | 328 (55.7%) | 2787 (52.7%) | 1126 (47.1%) | < 0.001 | 249 (54.6%) | 2283 (52.2%) | 913 (46.4%) | < 0.001 |
| Female | 184 (44.1%) | 1862 (48.7%) | 947 (54.8%) |  | 261 (44.3%) | 2499 (47.3%) | 1264 (52.9%) |  | 215 (45.4%) | 2087 (478%) | 1054 (53.6%) |  |
| **Child’s ethnicity** |  |  |  |  |  |  |  |  |  |  |  |  |
| White | 391 (96.5%) | 3697 (98.7%) | 1661 (98.2%) | 0.004 | 537 (95.7%) | 4896 (96.6%) | 2203 (95.6%) | 0.095 | 447 (97.2%) | 4222 (98.6%) | 1897 (98.3%) | 0.286 |
| Non white | 14 (3.5%) | 49 (1.3%) | 30 (1.8%) |  | 24 (4.3%) | 174 (3.4%) | 102 (4.4%) |  | 13 (2.8%) | 58 (1.4%) | 33 (1.7%) |  |
| **Parity** |  |  |  |  |  |  |  |  |  |  |  |  |
| 0 | 180 (44.2%) | 1748 (47.3%) | 801 (48.4%) | 0.002 | 247 (43.7%) | 2330 (45.8%) | 1089 (47.6%) | 0.004 | 204 (44.3%) | 1977 (46.8%) | 906 (48.1%) | 0.094 |
| 1 | 144 (35.4%) | 1332 (36.1%) | 589 (35.6%) |  | 199 (35.2%) | 1823 (35.9%) | 789 (34.9%) |  | 165 (35.9%) | 1519 (36.0%) | 660 (35.0%) |  |
| 2+ | 83 (20.4%) | 612 (16.%) | 266 (16.0%) |  | 119 (21.1%) | 932 (59.3%) | 409 (17.5%) |  | 91 (19.8%) | 726 (17.2%) | 319 (16.9%) |  |
| **Breastfeeding duration** |  |  |  |  |  |  |  |  |  |  |  |  |
| Never | 76 (19.5%) | 735 (20.0%) | 282 (17.0%) | 0.078 | 123 (22.7%) | 1189 (23.7%) | 462 (20.5%) | 0.005 | 90 (20.1%) | 919 (21.9%) | 370 (19.6%) | 0.036 |
|  | 88 (22.6%) | 840 (22.9%) | 360 (21.7%) |  | 132 (24.3%) | 1169 (23.3%) | 492 (21.8%) |  | 107 (23.9%) | 954 (22.7%) | 401 (21.2%) |  |
| 3–5 months | 60 (15.4%) | 635 (17.3%) | 314 (18.9%) |  | 79 (14.5%) | 852 (17.0%) | 408 (18.1%) |  | 61 (13.6%) | 724 (17.2%) | 346 (18.3%) |  |
| 6 months + | 166 (42.6%) | 1462 (39.8%) | 704 (42.4%) |  | 209 (38.5%) | 1808 (36.0%) | 869 (39.7%) |  | 189 (42.3%) | 1602 (38.2%) | 772 (40.9%) |  |
| **Family Adversity Index** |  |  |  |  |  |  |  |  |  |  |  |  |
| No adversity | 154 (36.9%) | 1645 (43.0%) | 784 (45.4%) | 0.023 | 205 (34.7%) | 2167 (41.0%) | 1018 (42.5%) | 0.015 | 167 (35.2%) | 1845 (42.2%) | 869 (44.2%) | 0.011 |
| Few adversities | 194 (46.5%) | 1657 (43.3%) | 727 (42.1%) |  | 284 (48.1%) | 2327 (44.0%) | 1035 (43.2%) |  | 233 (49.1%) | 1913 (43.7%) | 840 (42.7%) |  |
| Many adversities | 69 (16.5%) | 521 (13.6%) | 217 (12.6%) |  | 101 (17.1%) | 797 (15.1%) | 341 (14.2%) |  | 75 (15.8%) | 618 (14.1%) | 259 (13.2%) |  |
| **Birthweight** |  |  |  |  |  |  |  |  |  |  |  |  |
| Low (< 2500g) | 27 (6.5%) | 214 (5.6%) | 98 (5.7%) | 0.472 | 34 (5.8%) | 287 (5.4%) | 140 (5.8%) | 0.826 | 25 (5.3%) | 232 (5.3%) | 111 (5.6%) | 0.705 |
| Normal (≥ 2500-4000g) | 334 (80.1%) | 3138 (82.1%) | 1440 (83.3% |  | 480 (81.4%) | 4368 (82.5%) | 1978 (82.6%) |  | 383 (80.6%) | 3607 (82.4%) | 1625 (82.6%) |  |
| High (≥ 4000g) | 56 (13.4%) | 472 (12.3%) | 190 (11.0%) |  | 76 (12.9%) | 638 (12.1%) | 276 (11.5%) |  | 67 (14.1%) | 537 (12.3%) | 232 (11.8%) |  |
| **Gestation** |  |  |  |  |  |  |  |  |  |  |  |  |
| Preterm (< 37 weeks) | 16 (6.2%) | 173 (7.2%) | 96 (9.0%) | 0.121 | 27 (4.6%) | 220 (4.2%) | 114 (4.8%) | 0.477 | 20 (4.3%) | 170 (3.9%) | 93 (4.6%) | 0.455 |
| Term (≥ 37 weeks) | 243 (93.8%) | 2221 (92.8%) | 968 (91.0%) |  | 556 (95.4%) | 5006 (95.8%) | 2254 (95.2%) |  | 450 (95.7%) | 4144 (96.1%) | 1857 (95.4%) |  |

**Supplementary Table 4**Child seafood consumption at age 7 years and suboptimal child outcomes (IQ at age 8 and Strengths and Difficulties Questionnaire scores at age 7 and 9 years in the ALSPAC study: unadjusted logistic regression models

| **Outcomes** | **Odds of suboptimal IQ or SDQ compared with the reference category (unadjusted)** | | | | | **n** |
| --- | --- | --- | --- | --- | --- | --- |
|  | **0 vs ≥ 190 (ref) g/week** | | **1-190 (ref) vs ≥ 190 (ref) g/week** | | **Test for trend: P** |  |
|  | **OR (95% CI)** | **P** | **OR (95% CI)** | **P** |  |  |
| **Cognition: IQ total and subscores at 8 years** |  |  |  |  |  |  |
| Verbal | 0.89 (0.68, 1.16) | 0.395 | 1.03 (0.90, 1.18) | 0.751 | 0.751 | 5997 |
| Performance | 0.98 (0.76, 1.27) | 0.890 | 1.04 (0.91, 1.20) | 0.536 | 0.795 | 5986 |
| Total | 1.90 (0.69, 1.18) | 0.901 | 1.02 (0.89, 1.17) | 0.768 | 0.748 | 5969 |
|  |  |  |  |  |  |  |
| **Behaviour: SDQ subscores at 7 years** |  |  |  |  |  |  |
| Prosocial | 1.65 (1.33, 2.05) | < 0.001 | 1.24 (1.10, 1.41) | < 0.001 | < 0.001 | 8326 |
| Hyperactivity | 1.35 (0.95, 1.90) | 0.093 | 1.11 (0.91, 1.36) | 0.296 | 0.876 | 8310 |
| Emotional | 1.37 (0.98, 1.92) | 0.068 | 1.07 (0.87, 1.30) | 0.534 | 0.886 | 8318 |
| Conduct | 1.31 (1.00, 1.71) | 0.055 | 0.97 (0.83, 1.13) | 0.671 | 0.294 | 8329 |
| Peer problems | 1.72 (1.25, 2.35) | < 0.001 | 1.11 (0.91, 1.36) | 0.301 | 0.006 | 8322 |
| Total behavioural | 1.53 (1.07, 2.17) | 0.020 | 1.09 (0.88, 2.17) | 0.436 | 0.050 | 8277 |
|  |  |  |  |  |  |  |
| **Behaviour: SDQ subscores at 9 years** |  |  |  |  |  |  |
| Prosocial | 1.48 (1.13, 1.94) | 0.005 | 1.31 (1.12, 1.53) | < 0.001 | < 0.001 | 6843 |
| Hyperactivity | 1.93 (1.24, 3.01) | 0.004 | 1.38 (1.04, 1.83) | 0.027 | 0.002 | 6840 |
| Emotional | 1.26 (0.89, 1.78) | 0.190 | 0.80 (0.65, 0.99) | 0.036 | 0.798 | 6829 |
| Conduct | 1.03 (0.69, 1.55) | 0.893 | 1.24 (1.00, 1.53) | 0.048 | 0.231 | 6839 |
| Peer problems | 1.65 (1.18, 2.30) | 0.004 | 1.15 (0.94, 1.42) | 0.178 | 0.009 | 6829 |
| Total behavioural | 1.57 (0.99, 2.40) | 0.058 | 1.24 (0.95, 1.62) | 0.108 | 0.037 | 6819 |

Reference: ≥190 g/week

Reference categories: Three highest quartiles of IQ (Optimal) or score Close to average/Slightly raised or lowered on SDQ (Optimal).

*A test for trend was made on the assumption that the three seafood intake categories were equally spaced.

See Methods for details of variables.

Correlation coefficient for maternal fish intake vs child fish intake all < -0.20.
